# The impact of digital payments on sports lottery consumption decisions based on the moderating role of the business environment

**DOI:** 10.3389/fspor.2025.1628010

**Published:** 2025-07-30

**Authors:** Xinxin Zhang, Lumin Liu, Tianpei Li

**Affiliations:** ^1^ School of Physical Education, Myongji University, Yongin, Republic of Korea; ^2^ School of Physical Education, Jeonbuk National University, Jeonju, Republic of Korea

**Keywords:** digital payments, sports lottery consumption decisions, business environment, moderating role, regression analysis

## Abstract

**Introduction:**

The purpose of this paper is to examine the relationship between digital payment and sports lottery consumption decisions and to explore the underlying mechanism.

**Methods:**

Based on panel data from 31 Chinese provinces spanning 2011–2023, this study employs a fixed-effects panel model and a moderation analysis to investigate the relationship between digital payment and sports lottery consumption decisions, along with the moderating role of the business environment.

**Results:**

Digital payment significantly contributes to sports lottery consumption decisions. Furthermore, the business environment significantly moderates the impact of digital payment on these decisions. In terms of regional heterogeneity, digital payment plays a significant role in enhancing lottery consumption decisions in central and western regions.

**Discussion:**

The development of digital payment not only directly promotes sports lottery consumption decisions but also indirectly does so by improving the business environment. Enhanced lottery consumption decisions contribute to expanding national public welfare fundraising, supporting the development of public sports, and advancing the high-quality development of the sports industry. Additionally, this study highlights the importance of leveraging the government's guiding role, fostering digital innovation, enhancing the synergy between digital payment and the business environment, and adhering to the principle of “one policy for one place” to facilitate the orderly and sustainable digital transformation of China's sports lottery sector.

## 1 Introduction

Data released by China's Ministry of Finance indicated that in 2022, the China Sports Lottery generated 67.747 billion yuan for the national public welfare fund through a total annual turnover of 276.522 billion yuan. Among the provinces, Jiangsu, Zhejiang, Shandong, and Guangdong each recorded sales exceeding 20 billion yuan, while Hebei, Anhui, Fujian, and Henan each surpassed 10 billion yuan in sales (1). The sports lottery constitutes a significant component of China's sports industry. It generates substantial public welfare funds, which not only provide critical financial support for the development of both mass and competitive sports but are also allocated to areas such as public welfare, education, and healthcare. These funds enhance the construction of the social security system and contribute to the advancement of a strong sporting nation (2). In recent years, the government has introduced various regulations aimed at fostering the healthy development of the sports lottery industry. These policies seek to optimize the structure of lottery offerings, diversify game types, improve the efficiency of public fund utilization, and better meet public demand. According to the 14th Five-Year Plan for Sports Development issued by the State General Administration of Sports, the sustainable development of the sports lottery depends on enhancing technological support, leveraging digitization, and strengthening strategic management, innovation leadership, and talent development to improve overall distribution and sales effectiveness (3).

With the widespread adoption of digital technologies such as the Internet, big data, and artificial intelligence, digital payments enhance the convenience and accessibility of sports lottery purchases, thereby attracting a larger pool of potential lottery participants. In the State Council's Policy Routine Meeting held on December 28, 2023, it was stated in the introduction of the “Regulations on the Supervision and Administration of Non-Bank Payment Institutions” that, “China's mobile payment penetration rate has reached 86%, ranking first in the world” (4). The China Internet Network Information Center (CNNIC) released the “China Internet Development Status Statistical Report,” revealing that the number of Internet users in China reached 1.092 billion, with an Internet penetration rate of 77.5%, and the number of network payment users reached 954 million (5). Therefore, in the context of the rapid development of the digital economy, the widespread adoption of digital payment channels, the security and stability of platforms, and user trust in digital payments effectively lower the barriers to purchasing sports lotteries, stimulate consumer willingness to purchase, promote more rational and controllable purchase behavior, and provide a strong foundation for realizing the public welfare attributes and social responsibility of sports lotteries, making digital payment an essential tool for promoting lottery consumption decision-making.

A favorable business environment plays a crucial role in the development of digital payment by improving policies and regulations, upgrading infrastructure, and strengthening market supervision. Simultaneously, an efficient business environment optimizes the development of the third-party payment industry chain, simplifies the payment process, enhances transaction efficiency, and provides consumers with a seamless purchasing experience. Furthermore, the widespread adoption of digital payment enhances both the modernization and transparency of the business environment while simultaneously boosting consumers' trust and willingness to engage in consumption (6). In conclusion, the virtuous cycle between these two factors not only lowers the consumption threshold but also facilitates the digital transformation of the sports lottery market.

In summary, although digital payment and lottery consumption are both popular research areas, few studies have systematically explored how digital payment specifically affects sports lottery consumption decisions and the moderating role of the business environment amid the ongoing digitization and dynamic business environment. This research gap not only hinders a deeper understanding of the drivers of consumer behavior, but also prevents the lottery industry from effectively responding to challenges and seizing opportunities in digital transformation. In view of this, this study explores the impact of digital payment on sports lottery consumption decisions and its internal mechanism of action, based on panel data from 31 Chinese provinces from 2011 to 2023, using fixed panel models and moderated effects models, with the aim of offering insights for the development of a robust sports industry.

## 2 Literature review

With the deepening of China's economic system reform, the former National Sports Commission began to explore the possibility of issuing a sports lottery as a means to raise funds for sports development. On April 5, 1994, the Sports Lottery Management Center of the former National Sports Commission was formally established, marking the beginning of China's sports lottery entering a phase of legal and standardized management (7). A review of existing literature reveals that scholars have primarily examined the factors influencing sports lottery consumption from both macro and micro perspectives.

From a macro perspective, scholars focus on the impact of factors such as unemployment rate, population aging, GDP, average wage, and urbanization rate on sports lottery consumption decisions. Haiyun Zhu et al. (8) demonstrate that in economically developed regions with higher per capita income, the province's economic base serves as a guarantee for sports lottery consumption, with education level being the dominant influencing factor. Blalock et al. (9) found that the unemployment rate negatively affects lottery sales. Li Kun (10) found that sports lottery sales are positively correlated with local per capita GDP, education level, and urbanization rate, based on an analysis of the influencing factors. Bai Yufei et al. (11) found that the level of population aging significantly negatively affects sports lottery sales. From a micro perspective, scholars have studied the impact of factors such as age, gender, occupation, and financial literacy on sports lottery consumption decisions. Wang Aifeng et al. (12) found that the consumption psychology of sports lottery consumers varies significantly across age groups, with higher monthly family income correlating with lower sports lottery consumption. Garret et al. (13) demonstrated that consumer income level has a highly significant positive effect on lottery sales. Seungyeon Cho (14) investigated the relationship between financial literacy and frequency of lottery consumption, finding that an increase in the development level of the financial sector significantly increased lottery players' consumption.

Since the onset of the 21st century, the integration of the Internet, big data, artificial intelligence, and the real economy has been profound, with the payment industry playing a crucial role in advancing new technologies, driving continuous improvements in its digitalization. From the perspective of digital payment and consumer behavior, recent years have seen widespread adoption of digital payment, which has led to changes in household consumption patterns and structure (15). On one hand, digital payment facilitates consumer transactions by reducing transaction costs and decision-making time, thus promoting higher consumption frequency (16). On the other hand, in terms of reducing consumption pain, digital payment demands less cognitive effort from consumers, alleviates payment discomfort, and enhances the overall consumption experience. Consequently, digital payments tend to encourage consumers to increase their consumption budget (17). From a digital finance perspective, the penetration of digital payment is reshaping consumption patterns in risky areas, particularly in lottery and gaming products. Digital finance, as the integration of Internet technology with traditional finance, extends beyond mere payment functions, embedding comprehensive capabilities in data processing, risk management, and consumption guidance within the sports lottery consumption system. Studies, including those by Li Guozheng et al. (18), have highlighted that digital finance not only improves payment efficiency but also enhances platforms’ ability to guide and influence lottery buyers through precision marketing, big data analysis, and intelligent recommendation systems. Additionally, the inclusive nature of fintech platforms has broadened the potential consumer base for sports lotteries. In rural and underdeveloped areas, where traditional financial infrastructure is lacking, the widespread adoption of mobile payments and digital credit tools has included previously marginalized groups in the sports lottery consumer market, driving changes in consumption structure and behavior (19), thus contributing to the sinking and structural diversification of the market. Additionally, the study found a correlation between digital credit tools (e.g., “chanting,” “borrowing”) and increased sports lottery consumption, with some buyers overspending through installment and overdraft payments (20), heightening the risk of lottery addiction and financial vulnerability.

The business environment, as a key indicator of marketization, rule of law, and facilitation in a region or country, has been widely studied in relation to economic growth, enterprise behavior, and consumer consumption (21, 22). A high-quality business environment can indirectly foster the development and application of digital payments through various mechanisms, thereby influencing consumption decisions. On one hand, the business environment offers institutional and technical support for digital payment platforms by optimizing market regulation, bolstering institutional safeguards, and enhancing infrastructure investments. For instance, Zhao Tongtong et al. (23) note that government-driven development of data centers and communication infrastructure can significantly improve the stability and availability of digital financial platforms, providing protection for online consumption behaviors, including sports lotteries. Additionally, the improvement of the business environment affects consumers' psychological expectations and trust. Han Liangliang et al. (24) found that the business environment significantly incentivizes entrepreneurship and residents' willingness to invest, serving as a core psychological driver for the acceptance and depth of digital payment use. In the context of digital payments, which are characterized by high information asymmetry and network externalities, a favorable policy environment can effectively alleviate consumers' concerns about risks such as fraud and information leakage. Furthermore, some studies suggest that the business environment moderates the strength of the relationship between digital payments and consumer decisions. For example, Zhang Cheng et al. (25) argue that in regions with more transparent and fair business environments, the impact of digital technology (especially payment methods) on transforming consumption structures is more pronounced. Therefore, within the context of this study, the business environment functions not only as an independent variable influencing consumer behavior but also as a “catalyst” for digital payment, warranting further investigation into its regulatory mechanisms.

In summary, relevant literature has explored digital payment and sports betting consumer decision-making, but there is still a significant academic gap in the research on the integration of digital payment and sports betting consumer decision-making, and its mechanism is still unclear. In view of this, this study, based on the provincial panel data from 2011 to 2023, explores the impact of digital payment on sports lottery consumption decision-making and its intrinsic mechanism of action by establishing a benchmark regression model, in order for it to provide lessons and references for the development of a strong sports nation.

Compared with previous studies, this paper makes several contributions. First, it connects digital payment with sports lottery consumption decisions, analyzing their relationship and enriching the understanding of the specific mechanisms through which digital payment influences consumer behavior. Second, it introduces the comprehensive business environment index into the research framework and conducts empirical analyses, thereby broadening the scope of factors considered in sports lottery consumption research. Third, it investigates inter-city differences in the impact of digital payment on sports lottery consumption from a heterogeneity perspective and provides reasonable explanations, further enriching the literature on regional disparities in digital financial behavior.

## 3 Theoretical analysis and hypothesis formulation

### 3.1 The influence of digital payment on sports lottery consumption decisions

Digital payment has allowed traditional finance to overcome geographical limitations. Characterized by low cost, high speed, and broad coverage, digital finance has significantly reshaped the financial landscape and played a key role in promoting inclusive finance, improving financial efficiency, and fostering innovation (26). According to the “dual-channel mental accounting theory” (27), digital payments, due to their non-cash nature, reduce the pain of payment and are often psychologically categorized as “non-daily” or “entertainment” expenses. This weakens rational control and enhances emotional impulses, leading consumers to make more impulsive purchasing decisions (28).

Furthermore, Chinese household consumption has been shifting from survival-based to hedonic consumption. Greater digitization in payment methods eliminates spatial and temporal constraints associated with traditional payment systems, thereby increasing convenience and encouraging more frequent participation in lottery purchases (29).

In addition, studies on digital financial inclusion suggest that broader coverage promotes consumer engagement, while depth of usage reflects actual individual access to financial services, offering consumers more convenient, transparent, and personalized options (30). Moreover, the digital attributes of financial transformation, which distinguish it from traditional financial inclusion, exert a distinct positive influence on consumer decisions (31). Digital payment systems offer consumers increased flexibility and control over their finances, expand the variety of available lottery products, and enhance interactivity, ultimately stimulating enthusiasm for lottery purchases.Based on the above, the following research hypothesis is proposed.

H1: Digital payment positively influences sports lottery consumption decisions.
H2: The breadth of coverage of digital payment has a promoting effect on sports lottery consumption decisions.
H3: The depth of digital payment usage facilitates sports lottery consumption decisions.
H4: The degree of digitalization in payment systems has a promoting effect on sports lottery consumption decisions.

### 3.2 The moderating effect of the business environment on the relationship between digital payment and sports lottery consumption decisions

The business environment reflects the soft power of a country's or region's economy, and improvements in the business environment can foster the development of digital payments, thereby enhancing the convenience and safety of sports lottery consumption and contributing to the expansion of market scale (21). According to Social Cognitive Theory, individual behavior stems not only from one's cognition and motivation but is also shaped by the external environment, social structure, and interactive processes. In the process of digital payments influencing sports betting consumption decisions, the business environment, as a key external factor, significantly moderates the effectiveness of this behavioral pathway by influencing consumers' behavioral expectations, self-efficacy, and perceptions of outcomes (32).

On the one hand, improvements in the business environment significantly promote the construction of the third-party payment industry chain, while the development of this industry chain further drives the advancement of digital payment technology. Recently, new forms of third-party payments, such as QR code payment and near-field communication technology, have emerged rapidly, making digital payments safer, faster, and more diversified, thus enhancing consumers' sense of self-efficacy and control over their use. This suggests that the optimization of the business environment can influence factors of consumer psychology and behavior (24). On the other hand, improvements in the business environment promote the enhancement of infrastructure and the optimization of the policy environment, which facilitate the development of digital financial inclusion and, in turn, encourage consumer decisions to adopt digital payment for sports lottery. It has been shown that the construction of infrastructure for postal and telecommunication services, the Internet, data centers, and other sectors lays the foundation for digital inclusive finance, enabling the flow of data to drive the movement of technology and capital, thus providing basic support for digital payments to promote consumption (33). In this process, consumers’ enhancement of digital payment capabilities and accumulation of practical experience further fosters their behavioral self-confidence and intentions to continue adopting digital payments, reflecting the cyclical process of behavioral outcomes on individual cognitive restructuring in Social Cognitive Theory.

Therefore, the scientific and rational layout and effective application of infrastructure play a fundamental role in advancing the development of digital payment technology. Simultaneously, the optimization of the policy environment provides clearer policy support, promotes market expansion, and encourages the application of digital payments in the sports lottery sector, further enhancing consumers' willingness to engage in sports lottery consumption (34). Based on these insights, the research hypothesis is proposed.

H5: The business environment positively moderates the relationship between digital payment and sports lottery consumption decisions.

**Figure 1:**
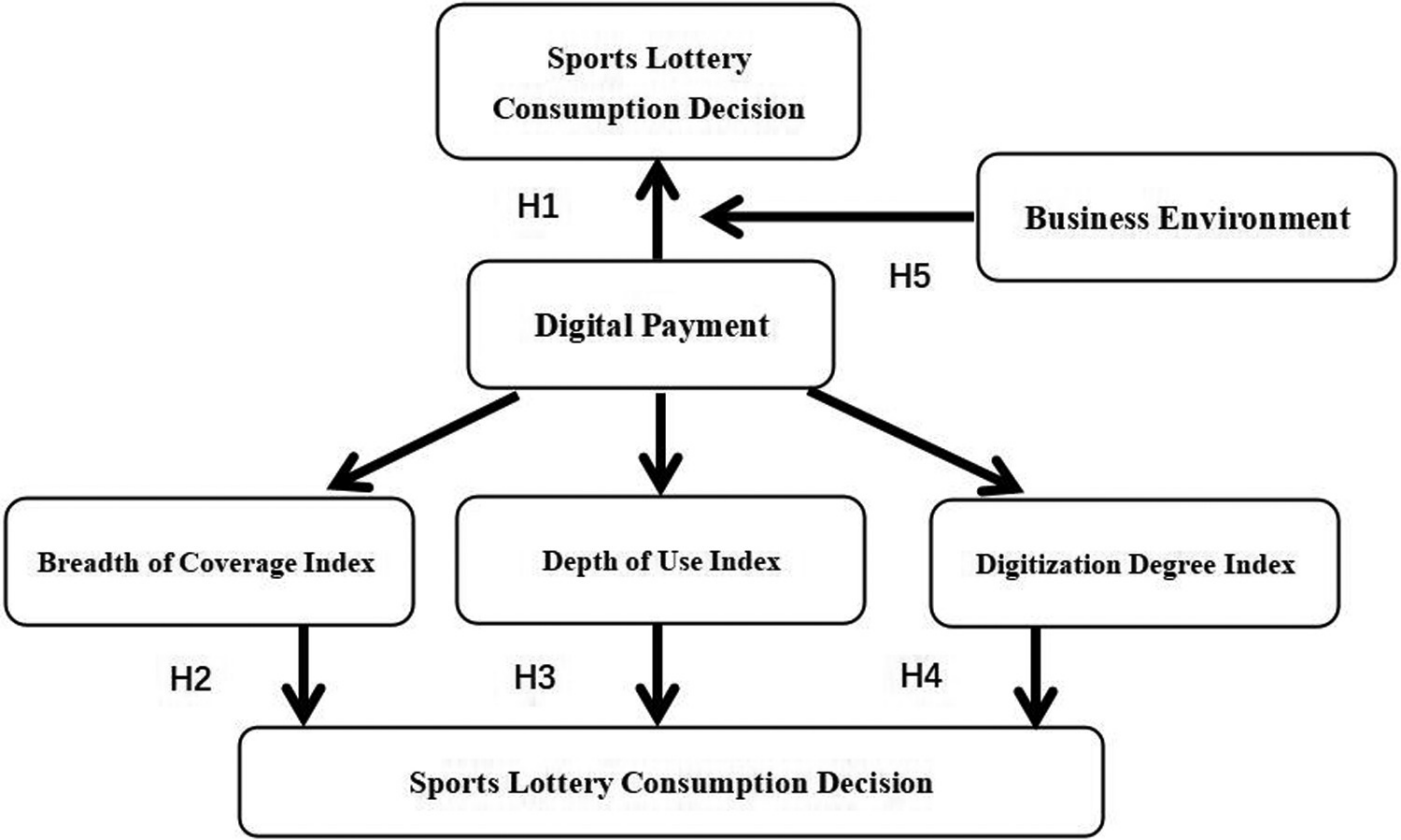
Path diagram of H1–H5 model.

In summary, according to the research hypothesis of this paper, the model path diagram of H1-H5 is constructed. As shown in Figure 1 below.

## 4 Research design

### 4.1 Variable selection

#### 4.1.1 Explained variable

The explained variable in this study is the sports lottery consumption decision (denoted as *INC*). Following the methodology of Liu Wenjing (35), this variable is measured by the ratio of per capita sports lottery consumption expenditure to per capita disposable income. This indicator reflects the relative level of sports lottery consumption in relation to individual income levels and serves as a reliable proxy for analyzing the decision-making behavior associated with sports lottery purchases.

#### 4.1.2 Core explanatory variables

The core explanatory variable in this study is digital payment. According to the *Research Report on Digital Payment and China's Inclusive Financial Development* jointly released by the China Inclusive Finance Institute of Renmin University of China and the Tencent Financial Research Institute, digital payment is recognized as a critical component of the modern financial system. It represents the integration of financial technology and is a key instrument in advancing digital financial inclusion. The development of digital payment systems and digital inclusive finance are thus considered mutually reinforcing.

To quantify digital payment, this paper employs the Digital Financial Inclusion Index (2011–2023) developed by the Peking University Center for Digital Finance (CDFI). This index is widely acknowledged for its scientific rigor and comprehensive representation of the development and spatial distribution of digital finance in China. It includes the following three first-level dimensions:

• DFI1: Breadth of Coverage Index – Captures the extent to which digital financial services are available across regions.
• DFI2: Depth of Use Index – Measures the intensity of digital financial services usage by individuals and enterprises.
• DFI3: Degree of Digitization Index – Reflects the technological advancement and digital transformation level of financial services.

These three sub-indices are used to capture the multidimensional impact of digital payment on sports lottery consumption decisions.

#### 4.1.3 Control variables

Drawing on the methodologies employed by previous scholars in related studies, this paper systematically identifies six control variables that are likely to significantly influence sports lottery consumption in China. These variables include the level of informatization, average wage, educational attainment, financial development, household consumption, and urbanization. The data for these variables were primarily obtained from the China Statistical Yearbook published by the National Bureau of Statistics and from the official websites of provincial finance bureaus (including those of autonomous regions and centrally administered municipalities). The study period spans from 2011 to 2023 to ensure that the findings are both contemporary and representative. This approach enhances the model's explanatory power and strengthens the verifiability and reproducibility of the findings (36).

(1) Informatization level (*Inform*). The improvement in informatization enhances the convenience, diversity, and real-time experience of sports lottery consumption, thereby influencing consumption decisions (37). This paper refers to Han Liangliang and Peng Yi (24) and adopts the percentage measure of the total postal and telecommunications business as a share of GDP.
(2) Average wage (*Wag*). The average wage level directly influences individuals’ disposable income. If the average wage in a region is higher, residents are generally more likely to have greater disposable income and may be more willing to allocate it to entertainment consumption, such as purchasing sports lottery tickets (30). This paper refers to Li Heng (38) and adopts the logarithmic measure of the average wage of employed workers.
(3) Education level (*Edu*). Individuals with higher education levels are more likely to engage in rational consumption and risk assessment, and may prefer more secure investment and consumption methods. Sports lottery betting is often associated with sports competitions, which require participants to possess certain knowledge. Therefore, the level of education may influence sports lottery consumption decisions (39). This paper refers to Li Ling and Zhang Ruilin (40) and adopts the logarithmic measure of the average number of students enrolled in higher education per 100,000 people.
(4) Financial development level (*finance*). Generally, an increase in financial development facilitates the acceptance of risks associated with purchasing sports lottery tickets. Therefore, the level of financial development may influence sports lottery consumption decisions. This paper refers to Fang Xianming (41) and Yang Qi (42) and adopts the value-added measure of the financial industry as a proportion of GDP.
(5) Consumption level of residents (*con*). Individuals with higher consumption levels are more likely to engage in additional entertainment consumption. As residents' consumption levels rise, an increasing number of individuals pay attention to sports competitions and view the purchase of sports lottery tickets as a form of entertainment associated with these competitions. This paper refers to Fang Xianming (41) and adopts the logarithm of the total consumption of social retail goods as the measure.
(6) Urbanization level (*urban*). An increase in urbanization is typically accompanied by the spread of modernization and urban culture, which may alter individuals' consumption concepts and behaviors, making them more receptive to new ideas, thus facilitating their understanding and purchase of sports lottery tickets. This paper refers to Li Ping (30) and others, Liu Wenjing (35), and Li Ling (40), and adopts the proportion of urban population to the year-end resident population as the measure.

#### 4.1.4 Moderating variable

The moderating variable in this study is the business environment (envir). This paper draws on the business environment index system developed by Yang Renfa (43) and colleagues, which includes four primary indicators and fifteen secondary indicators covering the macroeconomic environment, market environment, infrastructure, and policy environment (Table 1). Following the approach of Sun Guofeng (44) and others, the entropy value method is used to objectively and accurately measure the business environment index across 31 provincial-level administrative regions in China (including municipalities directly under the central government and autonomous regions) from 2011 to 2020.

**Table 1:** Business environment index system.

Primary indicator	Secondary indicator	Indicator description	Attribute
Macroeconomic Environment	Per capita GDP	Gross domestic product per capita	Positive
	Average wage level	Average wage of employees	Positive
	Consumption rate	Final consumption expenditure as a percentage of GDP	Positive
	Per capita fixed asset investment	Fixed asset investment per resident	Positive
	GDP growth rate	Annual growth rate of GDP	Positive
Market Environment	Foreign trade dependence	Total imports and exports as a share of GDP	Positive
	Total factor productivity (TFP)	A measure of overall production efficiency	Positive
	Number of employed persons	Number of employed persons at year-end	Positive
	Financing constraints	Loans by banking financial institutions as a share of GDP	Negative
Infrastructure	Urban road area per capita	Urban road area per resident	Positive
	Hospital bed availability	Number of beds in medical institutions	Positive
	Power supply capacity	Total electricity consumption	Positive
	Freight volume	Total freight volume	Positive
Policy Environment	Government intervention	Local fiscal expenditure as a share of GDP	Positive
	Corporate tax burden	Taxes and surcharges on main business/Total profit	Negative

First, all secondary indicators are normalized using the extreme value method to obtain standardized values *X_ij_*. Then, the entropy value method is applied to compute the business environment index for each region. The calculation formula is as follows (Equations 1–4):

(1) $$P_{ij}=\frac{X_{ij}}{\sum_{i=1}^{n}\;X_{ij}}$$

(2) $$e_{j}=-\frac{1}{\ln n}\overset{n}{\underset{i=1}{\sum }}\;P_{ij}\ln(P_{ij})$$

(3) $$\;g_{j}=1-e_{j}$$

(4) $$w_{j}=\frac{g_{j}}{\sum_{\;j=1}^{m}\;g_{j}}$$

Where *P*_ij_ is the ratio of the indicator value for calculating the *i* evaluated object on the *j* evaluation indicator; *e_j_* is the entropy value of the *j* indicator, and *n* is the number of indicators; *g_j_* denotes the information entropy redundancy; and *w_j_* is the weight of the *j* indicator.

### 4.2 Construction of empirical model

To test hypotheses H1, H2, H3, and H4, this study constructs Equation 5 to examine the impact of digital payment on sports lottery consumption. If hypothesis H1 holds, we expect the regression coefficient (*α*1) of the Digital Financial Inclusion Index (DFI) to be significantly greater than 0, indicating that digital payment has a positive effect on sports lottery consumption. The model also incorporates several control variables, including informatization level (Inform), average wage (Wag), education level (Edu), financial development level (Finance), residents' consumption level (Con), and urbanization level (Urban). Additionally, Year serves as a time-fixed effect to control for unobserved time factors that could impact sports lottery consumption decisions, *θ* represents fixed effects for regions, and *ε* is the random error term.

(5) $$\begin{array}{c} \mathrm{IN}C_{\mathrm{it}}=\alpha_{0}+\alpha_{1}\mathrm{DF}I_{\mathrm{it}}+\alpha_{2}\mathrm{Infor}m_{\mathrm{it}}+\alpha_{3}\;\ln\;\mathrm{Wa}g_{\mathrm{it}}+\alpha_{4}\;\ln\;\mathrm{Ed}u_{\mathrm{it}}\\ \;+A_{5}\ln\;\mathrm{financ}e_{\mathrm{it}}+\alpha_{6}\mathrm{co}n_{\mathrm{it}}+\alpha_{7}\mathrm{urba}n_{\mathrm{it}}+\mathrm{Yea}r_{t}+\theta_{i}+\varepsilon_{\mathrm{it}} \end{array}$$

In order to examine the moderating effect of the business environment on the relationship between digital payment and sports lottery consumption decisions, this study constructs Model 6, which includes an interaction term DFI × envir, capturing the combined influence of digital payments and the business environment.

(6) $$\mathrm{IN}C_{\mathrm{it}}=\beta_{0}+\beta_{1}\mathrm{DF}I_{\mathrm{it}}+\beta_{2}\mathrm{DF}I_{\mathrm{it}}\times \mathrm{envir}+\beta_{3}\mathrm{envi}r_{\mathrm{it}}+\beta_{j}\mathrm{contro}l_{\mathrm{it}}+\mathrm{Yea}r_{t}+\theta_{i}+\varepsilon_{\mathrm{it}}$$

## 5 Analysis of empirical results

### 5.1 Data sources and descriptive statistics

This study conducts empirical analysis using panel data from 31 provinces, municipalities directly under the central government, and autonomous regions in China from 2011 to 2023. The data for digital financial inclusion, representing the level of digital payment, is sourced from the Digital Financial Inclusion Index (2011–2023) compiled by Peking University. Other data is obtained from the Ministry of Finance of the People's Republic of China, the China Statistical Yearbook, and the official websites of Finance Bureaus across provinces.

To address potential heteroskedasticity in the regression process, natural logarithms are applied to the following variables: sports lottery consumption decision, the total digital inclusive finance index, the breadth-of-coverage index (DFI1), the depth-of-use index (DFI2), the digitization degree index (DFI3), average wage, education level, and residents' consumption level.

As shown in Table 2, the mean value of sports lottery consumption decision is 0.0044, while the mean value of the Digital Financial Inclusion Index (DFI) is 2.3365. The mean values for the DFI subcomponents are 2.2856 for breadth of coverage (DFI1), 2.3245 for depth of use (DFI2), and 2.4470 for digitization degree (DFI3). Observing the standard deviations, maximum, and minimum values of the variables, it is apparent that there are significant differences in residents' consumption levels and education levels across Chinese provinces. These differences are largely due to the wide geographical coverage of the sample area and the substantial regional variation in resources.

**Table 2:** Descriptive statistics results.

Variable name	Observations	Standard deviation	Minimum value	Maximum value	Mean
Sports Lottery Consumption Decision	403	0.0025	0.001	0.0239	0.0044
Digital Financial Inclusion Index (DFI)	403	0.2908	1.2101	2.68	2.3365
Breadth of Coverage Index (DFI1)	403	0.3599	0.2923	2.67	2.2856
Depth of Use Index (DFI2)	403	0.2792	0.8299	2.71	2.3245
Digitization Degree Index (DFI3)	403	0.2836	0.8797	2.68	2.447
Informatization Level (Inform)	403	0.073	0	0.391	0.065
Average Wage (Wag)	403	0.172	4.5058	5.382	4.8588
Education Level (Edu)	403	0.1349	0.0027	3.0342	3.4338
Financial Development Level (Finance)	403	0.0394	0.0265	0.4243	0.0739
Residents’ Consumption Level (Con)	403	0.7569	0	10.0721	7.7847
Urbanization Level (Urban)	403	0.1342	0.0027	0.9377	0.5907

### 5.2 Benchmark regression

Table 3 reports the results of the benchmark regression analysis, where sports lottery consumption decision is the dependent variable, and digital payment serves as the core explanatory variable. The model also includes control variables such as informatization level, average wage, education level, financial development level, consumption level, and urbanization level. Specifically, Model INC(1) shows the results without control variables, Model INC(2) presents results with control variables, and Models INC(3), INC(4), and INC(5) display the results for the breadth-of-coverage index, depth-of-use index, and digitization degree index under the digital inclusive finance index.

**Table 3:** Benchmark regression results.

Variable	*INC* (1)	*INC* (2)	*INC* (3)	*INC* (4)	*INC* (5)
DFI	0.0078***	0.0066***			
	(4.96)	(4.05)			
DFI1			0.0022***		
			(3.30)		
DFI2				0.0047***	
				(3.78)	
DFI3					−0.0006
					(−0.51)
Inform		0.0012	0.0010	0.0011	0.0006
		(0.89)	(0.77)	(0.87)	(0.47)
Wag		0.0090**	0.0090**	0.0103***	0.0108***
		(2.39)	(2.37)	(2.76)	(2.84)
Edu		0.0050***	0.0055**	0.0063***	0.0068***
		(2.61)	(2.87)	(3.43)	(3.52)
Finance		0.0017	0.0015	0.0018	0.0021
		(0.56)	(0.51)	(0.61)	(0.70)
Con		−0.0002	−0.0002	−0.0001	−0.0002
		(−1.22)	(−1.30)	(−1.05)	(−1.19)
Urban		−0.0030	−0.0028	−0.0027	−0.0022**
		(−1.52)	(−1.40)	(−1.34)	(−1.06)
Year	Yes	Yes	Yes	Yes	Yes
*θ*	Yes	Yes	Yes	Yes	Yes
*R* ^2^	0.1889	0.1800	0.2124	0.1371	0.1878
*F*	26.65***	21.75***	21.15***	21.52***	19.99***
*N*	403	403	403	403	403

*t*-statistics in parentheses, ***, **, and * indicate 1%, 5%, and 10% significance levels, respectively, below.

In Models 1 and 2, the regression coefficients of the core explanatory variable, digital payments (DFI), are 0.0078 and 0.0066, respectively. Both coefficients are significantly greater than zero and pass the 1% significance level test. This indicates that digital payments have a significant positive effect on sports lottery consumption decisions, regardless of whether control variables are included. The results suggest that an increase in the level of digital payments promotes sports lottery consumption decisions in the region. Digital payment systems provide consumers with greater freedom and convenience in making payments. Users no longer need to visit physical stores to make purchases; they can buy anytime and anywhere through mobile applications or online platforms, saving both time and energy (45). Furthermore, digital payment platforms offer a more diversified range of sports lottery products, which can attract users through push notifications and promotional activities, further boosting sports lottery consumption and enhancing the interactivity between consumers and the sports lottery market. Additionally, digital payment platforms typically feature user-friendly interfaces and transaction histories, allowing users to easily access their past transaction records, thereby improving payment security and enhancing user confidence and control when purchasing sports lottery tickets. Thus, research hypothesis H1 is validated.

In Models 3, 4, and 5, the regression coefficients corresponding to the core explanatory variables—breadth of coverage index (DFI1), depth of use index (DFI2), and digitization degree index (DFI3)—are 0.0022, 0.0047, and −0.0006, respectively. Models 3 and 4 pass the 1% significance level test. These results suggest that both the breadth of coverage and the depth of use have a significant positive impact on sports lottery consumption decisions. In contrast, the degree of digitization shows a negative but statistically insignificant effect, indicating that its relationship with sports lottery consumption decisions is not clearly evident. At this stage, the expanded breadth of digital payment coverage enables more individuals—including those in rural and remote areas—to access digital platforms conveniently, thereby broadening the potential customer base for sports lottery purchases. Greater depth of use has fostered familiarity and trust in digital payment systems, encouraging more consumer engagement—including purchasing sports lottery tickets—and thereby contributing to the healthy development of the sports lottery market (46). The insignificant relationship between digitization and lottery consumption may be attributed to regional heterogeneity, including disparities in informatization, income, education, and digital infrastructure penetration, which in turn influence consumer acceptance and frequency of digital service usage, ultimately weakening the impact of digitization on lottery decisions (47).

According to Model 2, the regression coefficients for the control variables—level of informatization and level of financial development—are 0.0012 and 0.0017, respectively, and do not reach statistical significance. This suggests that while informatization exerts a positive effect on sports lottery consumption decisions, the effect is not statistically significant. The regression coefficients for average salary and education level are 0.0090 and 0.0050, respectively, and pass the 5% and 1% significance tests, indicating that these two factors significantly and positively influence sports lottery consumption decisions. The coefficients for residents’ consumption level and urbanization level are −0.0002 and −0.0030, respectively, and both fail to pass the 10% significance threshold, indicating negative but statistically insignificant effects.

In summary, both informatization and financial development exhibit positive but insignificant effects on sports lottery consumption decisions. This may be because, although higher informatization enhances access to digital payment tools and lottery-related information, psychological factors such as personal interest and risk preference may play a more dominant role, thereby diluting the measurable impact. While financial development enhances payment convenience and fund liquidity, it primarily caters to investment and savings needs. Its stimulative effect on occasional or recreational consumption, such as sports lottery participation, remains limited and thus statistically insignificant.

Conversely, education level and average salary exert significant positive effects on sports lottery consumption. This may be attributed to the fact that more educated consumers are better able to comprehend the rules and public welfare functions of sports lotteries, thereby increasing their acceptance. Moreover, rising average salaries increase disposable income, enabling consumers to allocate more resources to recreational or non-essential consumption after fulfilling basic needs (48). Additionally, individuals with higher income and education levels are more likely to diversify their consumption patterns, further encouraging the purchase of sports lottery tickets.

Finally, residents' consumption level and urbanization show negative yet statistically insignificant effects, possibly because these factors, although indicative of improved living standards, do not directly influence demand for sports lottery consumption (49). Although overall consumption reflects greater purchasing power, growth is often concentrated in essential or high-frequency goods, which contributes minimally to recreational expenditures like lottery purchases. Similarly, although urbanization enhances the consumption environment, it also promotes access to diverse entertainment options, thereby reducing the relative appeal of sports lotteries.

### 5.3 Robustness tests

#### 5.3.1 Endogeneity test

There are two potential sources of endogeneity in examining the impact of digital payment on sports lottery consumption decisions. The first is the omitted variable problem, where unobservable variables might affect the relationship between digital payments and sports lottery consumption, leading to errors in the regression results. The second is the issue of mutual causality, where sports lottery consumption is not a basic necessity but rather a reflection of the improvement in residents' quality of life. In the current context, as consumer preferences diversify, residents are more likely to embrace new consumption methods, such as using digital payments for sports lottery purchases. This creates a potential reverse causality problem.

To address these endogeneity concerns, this paper follows Shi Xiaokun (50) and uses Internet penetration rate (HLW) as an instrumental variable.

The results from using two-stage least squares (2SLS) to handle the endogeneity problem are presented in Table 4. First, in terms of instrumental variable testing, the Kleibergen-Paap rk LM statistic yields a *p*-value of less than 0.01, which rejects the null hypothesis of non-identifiability. Moreover, the Kleibergen-Paap rk Wald F-statistic and the Cragg-Donald Wald F-statistic both exceed the Stock-Yogo critical value at the 10% level (12.97), rejecting the null hypothesis of weak instruments.

**Table 4:** Results of endogeneity test.

Variable	*INC* (6)	
	Phase I	Phase II
DFI		0.0025**
		(3.52)
HLW	0.0059**	
	(4.12）	
inform	−0.0076*	0.0025**
	(−1.07)	(2.13)
Wag	−0.0063*	−0.0054
	(−0.95)	(−1.57)
Edu	−0.0003	0.0027**
	(−1.64)	(1.93)
finance	0.0197*	−0.0058
	(1.57)	(−0.33)
con	−0.003	−0.0001*
	(−0.67)	(−2.36)
urban	0.0017	−0.0025
	(0.55)	(1.87)
constant term	0.0487**	−0.035**
	(5.11)	(3.55)
year	YES	YES
R^2^	0.1263	0.2642
Phase I F-value	17.35***	
Kleibergen-paap rk LM statistic	23.05***	
kleibergen-paap rk wald F statistic	18.73***[12.97]	
N	403	403

Values in parentheses are critical values at the 10% level of the Stock-Yogo test.

Second, the benchmark regression results from the second stage, after addressing potential endogeneity issues, maintain the same direction and pass the 5% significance test. Therefore, the assumptions in this paper are robust.

#### 5.3.2 Other robustness tests

To further verify the robustness of the benchmark regression results, this paper conducts additional robustness checks by adjusting the sample size and applying tail reduction. The outcomes are presented in Table 5. Model (8) reports the regression results after excluding the samples from Beijing, Tianjin, Shanghai, Chongqing, and the Tibet Autonomous Region (TAR). This exclusion is based on two considerations: first, the economic data of TAR differ significantly from other provinces, which could introduce bias into the analysis; second, municipalities are often the first to implement pilot economic policies, potentially affecting the generalizability of the results. After removing these regions and re-running the regression, the coefficient of digital payments remains significantly positive, indicating that the main conclusion holds even after adjusting for these influential samples. Model (9) addresses potential distortion caused by outliers. To mitigate the impact of extreme values on the benchmark regression results, all variables are winsorized at the top and bottom 1%. After this treatment, the revised dataset is used for regression analysis. The results show that the coefficient of digital payment continues to pass the 1% significance test, further confirming the stability and reliability of the findings.In summary, the conclusions remain consistent under both robustness test strategies, reinforcing the validity of H1.

**Table 5:** Robustness test results.

Variable	*INC* (8)	*INC* (9)
DFI	0.0035***	0.0027***
	(3.40)	(3.22)
Inform	0.0017**	0.0016**
	(2.40)	(2.05)
Wag	−0.0047**	−0.0035*
	(−1.98)	(−1.31)
Edu	0.0077***	0.0054*
	(5.89)	(3.44)
Finance	0.0024	0.0020
	(1.51)	(0.87)
Con	−0.0002**	−0.0002*
	(−2.29)	(−2.28)
Urban	−0.0008	−0.0010
	(−0.71)	(−1.14)
Year	Yes	Yes
*θ*	Yes	Yes
*R* ^2^	0.2350	0.1928
*F*	55.97***	28.72***
*N*	338	403

### 5.4 Mechanism analysis

#### 5.4.1 Analysis of moderating effects

The coefficient of the interaction term provides insight into both the direction and significance of the moderating effect. To examine whether the business environment moderates the relationship between digital payment and sports lottery consumption decisions, this study applies Equation (6), with a particular focus on the interaction term between the business environment and digital payment. The results are presented in Table 6.

**Table 6:** Results of moderating effects analysis.

Variable	*INC* (10)
DFI	0.0029
	(1.47)
Envir	−0.0370***
	(−3.42)
DFI × envir	0.0151***
	(3.53)
Control variable	Yes
Year	Yes
*θ*	Yes
*R* ^2^	0.1437
*F*	20.92***
*N*	403

The findings indicate that the regression coefficients of the interaction terms are significantly positive at the 1% level, suggesting that the business environment positively moderates the impact of digital payment on sports lottery consumption. In other words, the presence of a favorable business environment amplifies the positive influence of digital payment on lottery consumption behavior.

Specifically, a sound business environment often reflects more robust digital infrastructure and technical support, which can enhance payment convenience and security. This reduces user concerns about digital transactions and thereby increases their willingness to engage in lottery purchases. Moreover, a well-regulated business environment helps to standardize digital payment systems, curtail illegal lottery activities, and protect consumer rights, further boosting consumer confidence in both digital payments and the sports lottery system (25). Thus, an improved business environment enhances the user experience of digital payments, strengthens trust, and fosters a more favorable consumption climate. Consequently, digital payment systems are more effective in promoting sports lottery consumption when supported by a strong business environment.

Based on these results, Hypothesis H5 is supported.

#### 5.4.2 Heterogeneity test

Given the disparities in economic and social development across different regions of China, it is essential to conduct in-depth regional analyses. Based on the classification standards of the National Bureau of Statistics, China's 31 provinces (including autonomous regions and municipalities directly under the central government) are divided into three regional sub-samples: the eastern region (Beijing, Tianjin, Hebei, Liaoning, Shanghai, Jiangsu, Zhejiang, Fujian, Shandong, Guangdong, and Hainan), the central region (Shanxi, Jilin, Heilongjiang, Anhui, Jiangxi, Henan, Hubei, and Hunan), and the western region (Inner Mongolia, Guangxi, Chongqing, Sichuan, Guizhou, Yunnan, Tibet, Shaanxi, Gansu, Qinghai, Ningxia, and Xinjiang). This classification enables the examination of regional heterogeneity in the impact of digital payment on sports lottery consumption decisions.

Table 7 shows that the regression coefficient for the eastern region is −0.0011, which is not statistically significant at the 10% level, while the central region has a coefficient of 0.0091, significant at the 5% level, and the western region has a coefficient of 0.0104, also significant at the 5% level. These results indicate that digital payment in the eastern region exerts a negative, though statistically insignificant, effect on sports lottery consumption decisions. In contrast, digital payment demonstrates a significantly positive impact on sports lottery consumption decisions in the central and western regions.

**Table 7:** Heterogeneity test results.

Variable	*INC*	*INC*	*INC*
	(Eastern)	(Central)	(Western)
DFI	−0.0011	0.0091**	0.0104**
	(−0.42)	(2.23)	(2.26)
Inform	0.0017	0.0022	0.0036
	(1.07)	(1.55)	(1.18)
Wag	0.0067	−0.0094***	0.0053
	(1.25)	(−2.79)	(0.63)
Edu	0.0061***	−0.0052*	0.0049
	(2.95)	(−1.74)	(0.98)
Finance	0.0057	−0.0023	0.0005
	(0.67)	(−0.27)	(0.12)
Con	−0.0002**	0.0005	0.0045
	(−1.99)	(1.43)	(1.37)
Urban	−0.0052**	−0.0008	−0.0245
	(−0.44)	(−0.69)	(−1.37)
Year	Yes	Yes	Yes
*θ*	Yes	Yes	Yes
*R* ^2^	0.3309	0.2685	0.0005
*F*	25.42***	23.65***	5.49***
*N*	143	104	156

In summary, the regional differences in the impact of digital payment on sports lottery consumption can be attributed to disparities in economic development, digital infrastructure, consumer behavior, and financial technology adoption. First, in terms of economic development, the eastern region is highly developed, with residents enjoying high disposable incomes and diversified, high-end consumption structures. By contrast, consumption in the central and western regions is still driven by basic and upgrading needs, making residents more responsive to accessible, low-threshold consumption activities such as sports lottery purchases (18). Second, disparities in digital infrastructure construction also play a critical role. According to the China Internet Network Information Center (CNNIC), while the national Internet penetration rate has reached 77.5%, coverage and terminal access remain insufficient in remote and rural areas (5). Recent government initiatives under the “digital countryside” policy have improved mobile payment access in these areas, thereby stimulating lottery consumption among previously underserved populations.

Third, the marginal utility of digital payment penetration appears to be diminishing in the eastern region, where usage is already highly saturated. This saturation limits further gains in perceived convenience, thereby weakening digital payment's impact on sports lottery behavior (51). Fourth, differences in financial literacy and trust in digital platforms also contribute to regional heterogeneity. In the central and western regions, efforts to promote digital financial education and build consumer trust have successfully brought lower-literacy groups into the digital payment ecosystem, thus enhancing their participation in the sports lottery market.

Therefore, the uneven development of regional economies and the varying pace of digital transformation jointly determine the asymmetric impact of digital payment on sports lottery consumption. Policymakers should thus adopt differentiated strategies, particularly in the central and western regions, by intensifying investment in digital infrastructure and promoting inclusive fintech development, thereby unlocking the full potential of digital lottery consumption.

## 6 Discussion

First, digital payment significantly influences sports lottery consumption decisions, with the breadth of coverage and depth of use serving as the primary drivers. This finding strongly supports the core hypotheses (H1, H2, and H3) proposed in this study, as framed by the dual-channel mental accounting theory. From a theoretical standpoint, the widespread availability and convenience of digital payment systems substantially reduce the psychological “pain” of purchase, enabling consumers to mentally allocate such purchases to their “entertainment expenditure” account. This weakens the inhibitory function of the rational system and enhances the emotion-driven propensity to consume (52). Moreover, the perceived usefulness (e.g., anytime-anywhere convenience, diverse product offerings) and perceived ease of use (e.g., intuitive operations, transparent transaction records) associated with digital payment increase consumers' perceived behavioral control over sports lottery purchasing, thereby lowering both the behavioral threshold and psychological resistance (29). These insights not only validate the relevance of existing consumer behavior theories in the context of non-essential, recreational consumption but also enrich our understanding of consumption dynamics in the digital economy. Although the direct effect of the degree of digitization has not yet reached statistical significance, the breadth and depth of digital payment adoption have emerged as critical enablers for activating the sports lottery market.

Second, the business environment significantly moderates the relationship between digital payment and sports lottery consumption decisions. This finding is consistent with Social Cognitive Theory (H5), which emphasizes the role of external environmental factors in shaping individual behavior. A favorable business environment—characterized by sound policies, robust infrastructure, and transparent regulatory frameworks—improves the perceived reliability and security of digital payment platforms. This, in turn, alleviates consumer concerns regarding risks such as information leakage or fraud and enhances trust in both digital payments and lottery purchases (53). In essence, a supportive institutional environment amplifies the efficacy of digital payments and strengthens their influence on consumer behavior. This observation provides valuable empirical evidence for understanding the interplay between macro-level institutional contexts and micro-level consumption behavior, highlighting the foundational role of institutional quality in the evolution of the digital economy.

Third, the impact of digital payment on sports lottery consumption exhibits significant regional heterogeneity. Specifically, the influence is statistically significant in the central and western regions, but not in the eastern region. This divergent pattern reflects the complex landscape of digital technology–enabled consumption within the broader context of China's uneven regional development. In the central and western regions, the increasing breadth and depth of digital payment adoption have effectively addressed gaps in traditional financial infrastructure, thereby expanding the potential consumer base. The convenience and accessibility of digital payments confer greater marginal utility in these less-developed areas, substantially encouraging sports lottery participation (54). Conversely, in the eastern region—where digital payment is already deeply embedded and the consumption structure has shifted toward high-end and diversified options—the marginal effect of digital payment on lottery consumption appears saturated or offset by alternative entertainment choices. Therefore, strategies to promote digital technologies and related industries must account for regional disparities in economic development, infrastructure maturity, consumer behavior patterns, and fintech acceptance (55).

## 7 Conclusions

To examine the effect of digital payment on sports lottery consumption decisions, this study analyzes panel data from 31 provinces in China spanning from 2011 to 2023 and investigates the underlying mechanisms. The findings reveal the following: First, digital payment significantly promotes sports lottery consumption decisions—both the expansion of digital payment coverage and the deepening of its usage have a strong positive impact. Second, the business environment plays a significant positive moderating role in the relationship between digital payment and sports lottery consumption decisions. Third, regional heterogeneity exists in the impact of digital payment on sports lottery consumption, with significant effects observed in the central and western regions.

## Data Availability

The raw data supporting the conclusions of this article will be made available by the authors, without undue reservation.
